# Extensive variability in the composition of immune infiltrate in different mouse models of cancer

**DOI:** 10.1186/s42826-020-00075-9

**Published:** 2020-11-19

**Authors:** Virginia Niemi, Douglas Gaskarth, Roslyn A. Kemp

**Affiliations:** Department of Microbiology and Immunology, University of Otago, PO Box 56, Dunedin, 9010 New Zealand

**Keywords:** Cancer, T cell, Colorectal, Surgery, Immune

## Abstract

Mouse models are invaluable tools for cancer immunology research. However, there are differences in the immune response to the tumour depending on the model used, and these differences are not often characterised on their own. Instead they are often only analysed in response to a therapeutic immune modulation. There are important issues with translatability into effective clinical research when considering the choice of mouse models. Here we analysed the tumour immune microenvironment and modified aspects of the tumour model to determine the effect on the composition of the immune infiltrate. Mice injected subcutaneously with the melanoma cell line, B16-OVA, had a higher frequency of T cells, especially CD8+ T cells, than mice injected subcutaneously with CT26 colorectal adenocarcinoma cells. We compared the same tumour cell line (CT26) delivered either subcutaneously and intracaecally. To minimise immunological impacts due to the invasive surgery procedure, we optimised an existing intracaecal injection protocol. Intracaecal tumours had a higher frequency of infiltrating CD3+ CD4+ T cells and a lower frequency of CD3-CD19- (putative NK cells) than subcutaneous tumours. In contrast, there was a higher frequency of F480+ macrophages in subcutaneous tumours than intracaecal tumours. These data demonstrate that variability between animals, between experiments and within tumour models, can lead to difficulty in interpreting the infiltrating immune response and translating this response to clinical research.

**Supplementary Information:**

The online version contains supplementary material available at 10.1186/s42826-020-00075-9.

## Introduction

There is a strong prognostic role for immune cell infiltrates into tumours [1]. In humans, the frequency of infiltrating T cells can be used to stage patient disease in colorectal cancer (CRC) more accurately than current histopathologic methods [2]. New immune-based therapies have had success in many human cancers, however, there are still a large proportion of patients who do not respond to these immune interventions, despite evidence from pre-clinical models of efficacy. The lack in translatability from mouse tumour models into humans raises questions about the variability in mouse models used by different investigators to study anti-tumour immune responses.

Animal models provide an excellent method to study the in vivo immune response to cancer [3]. They allow in depth investigation of the tumour microenvironment (TME) to identify mechanisms of immune protection, biomarkers of disease progression and potential new immunotherapeutic targets. In this study, we analysed the variability in the immune infiltrate of tumours in different mouse models. We highlight the variability and heterogeneity of immune responses between models and within experiments. We first compared two commonly used tumour models – B16 (transfected with ovalbumin (OVA)), a melanoma cell line, in C57Bl/6 mice and CT-26, a colorectal adenocarcinoma, in Balb/c mice. We show that the immune response in the lymphoid organs and the tumour is different when mice are challenged subcutaneously with B16-OVA versus CT26, emphasising the need to select a consistent and representative model. Second, we show that the frequencies of tumour-infiltrating myeloid and lymphoid cells are different in mice challenged with CT26 either intracaecally or subcutaneously, highlighting the need to study physiologically relevant sites. Finally, we demonstrate extensive variability both between and within experiments in mouse models of cancer.

## Methods/experimental

### Tumour cell culture

CT26 cells (ATCC, VA, USA) were cultured in complete Roswell Park Memorial Institute (RPMI) medium (Gibco, NY, USA) supplemented with 10% foetal calf serum (FCS, PAA laboratories, Morningside, Australia), 1% Penicillin/Streptomycin (Life Technologies, Carlsbad, CA, USA), and 0.1% 2-mercaptoethanol (Life Technologies) at least 2 days prior to subcutaneous tumour injection or intracaecal tumour injection. B16-OVA cells (Malaghan Institute of Medical Research, Wellington, New Zealand) were cultured in the same conditions with the addition of 100 mL geneticin (Invitrogen, Carlsbad, CA, USA). Cells were approximately 80% confluent before harvest.

### Subcutaneous tumour injection

All mouse work was performed inside a class II biological safety hood. Mice were injected subcutaneously with 100 μL of the cell suspension (B16-OVA into C57Bl/6 J mice and CT26 into Balb/c mice) into the left flank using a 26-gauge needle. Control mice were injected with 100 μL phosphate buffered saline (PBS, Invitrogen) in the left flank. After injection, mice were returned to their cages and monitored on days 1, 5, 7, 12, 14, and 17 for animal well-being and tumour growth. Once tumours reached approximately 3 mm in diameter, as measured by callipers, mice were culled (day 17–18).

### Intracaecal surgery

Cells were harvested and resuspended in sterile PBS at a concentration of 1 × 10^6^ cells/mL in 25 mL. Surgical equipment was set-up inside a biological safety cabinet (Fig. 2b). Mice received preoperative painkiller (temgesic, 0.1 mg/kg, Animal Welfare Office (AWO), University of Otago), antibiotic (amphoprim, 30 mg/kg, AWO), anti-inflammatory (carprofen, 5 mg/kg, AWO), and saline solution subcutaneously. Balb/c mice were anaesthetised via isoflurane before transfer to a nose cone for the remainder of the procedure. Eye gel was applied, the abdomen shaved, and the surgical site cleared of fur. Local anaesthesia (marcain, 1.9 mg/kg, AWO) was injected subcutaneously near the surgery site. Avagard (Capes Medical, Tauranga, New Zealand) was used to disinfect the surgery site and the mouse covered with a sterile drape.

Slightly right of the midline, a 10 mm incision was made in the skin and peritoneum. The caecum was externalised, before being placed on pre-cut, PBS-moistened sterile gauze. The microscope was focused on the caecum before injection of 25 μL of CT26 cells into subserosa of the blind-ended pouch of the caecum. The caecum was moistened with PBS and returned to the abdominal cavity. Both the abdominal wall and skin incisions were closed with 5–0 sutures using 3–4 and 4–5 simple interrupted stitches. The wound was cleaned with sterile PBS and the mouse placed in a heated recovery cage. The mouse was monitored every 5 min until it regained consciousness. Mice were monitored for recovery 1, 4, and 24 h after surgery and daily for 5 days post- surgery. This included weighing the mice and checking for signs of pain and infection. Mice were then monitored twice weekly until sacrifice (15 days post-surgery).

### Tissue processing

Mice were euthanised via CO_2_ inhalation. Death was confirmed by cervical dislocation. Spleens, lymph nodes and tumours were removed, placed in 1–2 mL of RPMI in a 6-well plate and kept on ice. Tissues were dissociated and resuspended in 1 mL of fluorescence activated cell sorting (FACS) buffer (PBS, 0.5% FCS, 0.01% sodium azide, Prolab, Geldenaakaeban, Germany) through a 70 μM filter. Large tumours were resuspended in 3 mL of FACS buffer through a 70 μM filter. For spleen samples, red blood cells were lysed (1.2% ammonium chloride, Sigma Aldrich, 0.1% potassium bicarbonate, Sigma Aldrich, Auckland, New Zealand, 0.03% Ethylenediaminetetraacetic acid, B&M GmBH, Germany) and cells resuspended in FACS buffer. Cell numbers from all tissue samples were counted using trypan blue exclusion. Unstained control and fluorescence minus one (FMO) controls for each antibody were prepared.

### Flow cytometry

Each sample or FMO was stained using Texas red live/dead dye (Life Technologies). Cells were incubated for 30 min on ice and in the dark. Live/dead stained cells were washed and resuspended in FACS buffer. Antibodies (Additional Files 1 and 2) were added and incubated for 30 min on ice in the dark, washed in FACS buffer, and resuspended in 1% paraformaldehyde (PFA; Sigma Aldrich) for 30 min. After surface staining, all samples, FMOs, and the unstained control were resuspended in PFA and incubated at room temperature, in the dark, for 30 min. Cells were then resuspended in FACS buffer. Compensation beads were prepared using One Comp eBeads (ThermoFisher Scientific, Waltham, MA, USA). Beads were incubated in the dark, on ice for 20 min before washing. Acquisition of events was performed on a LSR-FORTESSA using FacsDIVA (version 8.0, BD-Biosciences). Data was exported as flow cytometry standard 3.1 files and analysed using FlowJo (version 10.0.7, Tree Star, Ashland, OR,USA) software.

## Results

### Comparison of immune responses in mice injected with two common tumour cell lines

**Fig. 1:**
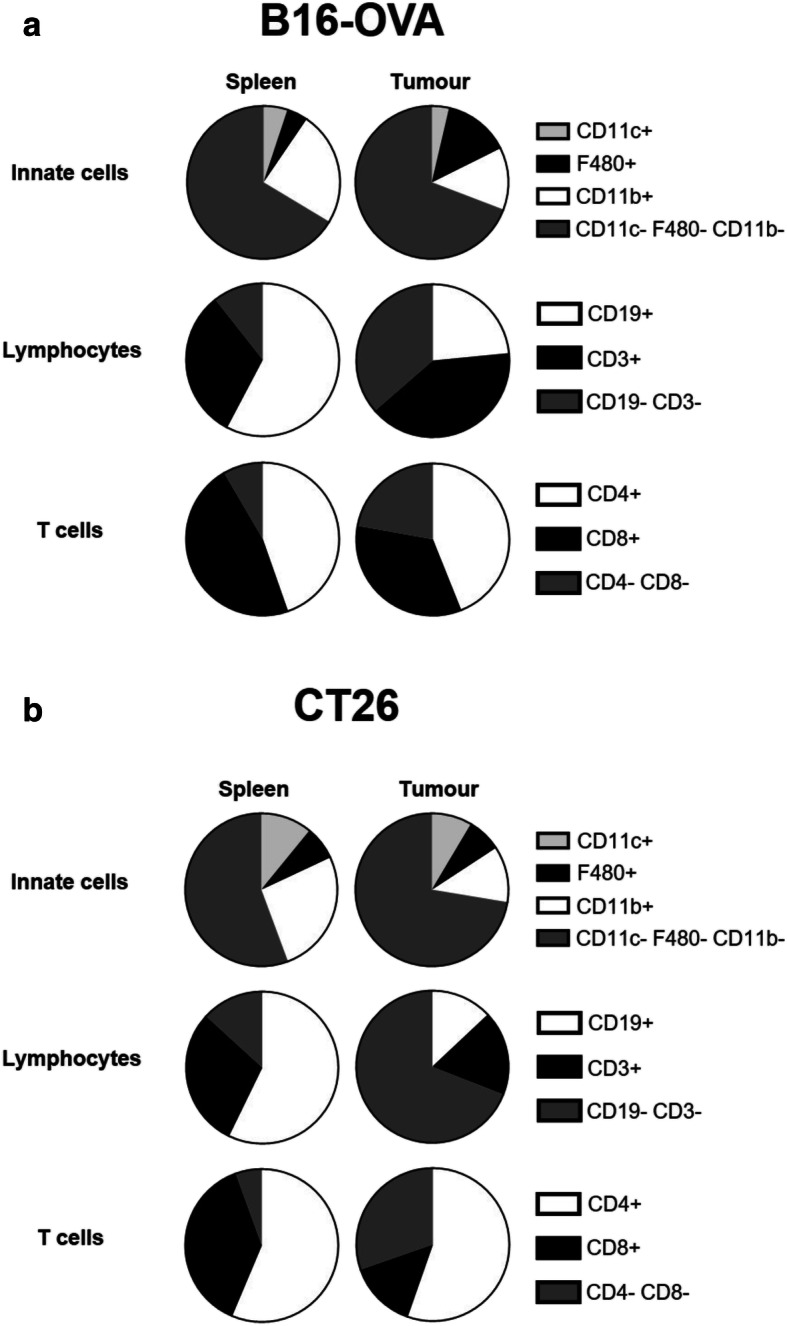
The immune response to C57Bl/6 mice given SC B16-OVA tumours compared to the immune response of Balb/c mice given SC CT26 tumours. Mice were challenged subcutaneously with B16-OVA or CT26 cells. Results are averaged from 3 (**a**, B16-OVA, *n* = 18–21) and 4 (**b**, CT26, *n* = 25) pooled experiments. Data are shown as the mean frequency of each subset as a percentage of live myeloid cells (CD11c+, F480+, and CD11b+, or CD11c- CD11b- F480-) or live lymphocytes (CD3+, CD19+, CD3- CD19-) or CD3+ T cells (CD4+, CD8+, CD4- CD8-) for all analysed mice

We initially compared the immune response of mice to two commonly used tumour models – a melanoma cell line, B16-OVA and a colorectal adenocarcinoma cell line, CT26, each administered subcutaneously. We calculated the frequency of cells from the myeloid and lymphoid compartments of the spleen, peripheral lymph nodes and tumour. Lymphoid cells were defined as CD19+ B cells, CD3+ T cells, CD19-CD3- cells in a lymphoid gate (see Additional File 3), and T cells were further divided into CD4+, CD8+ and CD4-CD8-. Double negative CD3+ T cells have previously been reported in mouse models of cancer [4] and are associated with enhanced tumour growth. Dendritic cells (CD11c/CD11b), F480+ macrophages, F480- macrophages, and other large cells are shown in the gating strategy in Additional File 4**.** Figure 1 shows the different frequencies of immune cells in organs from mice injected with B16-OVA (Fig. 1a) and CT26 (Fig. 1b). As expected, the frequencies of different immune cells in the spleen were similar between the two groups, representing a standard systemic response. This was also true of the lymph nodes (data not shown). However, there was a different profile of lymphocytes infiltrating the tumour in mice injected with CT26 versus those injected with B16-OVA. There was a higher frequency of T cells in B16-OVA tumours than in CT26 tumours, and of those T cells, a higher frequency of CD8+ T cells in B16-OVA tumours than CT26 tumours. There was a higher frequency of CD3-CD19- cells in CT26 tumours compared to B16-OVA tumours; these may represent NK cells, although these were not specifically studied in this study.

These data highlight the variability seen in two mouse strains injected subcutaneously with two different tumour cell and cautions against making generalised statements about cancer immune responses. The results emphasise the importance of testing immunological therapeutics and investigating immune cell dynamics in a variety of models, as differences in mouse strain alone have the potential to alter the interpretation of results.

### Development of an intracaecal surgical mouse model to study colorectal cancer

**Fig. 2:**
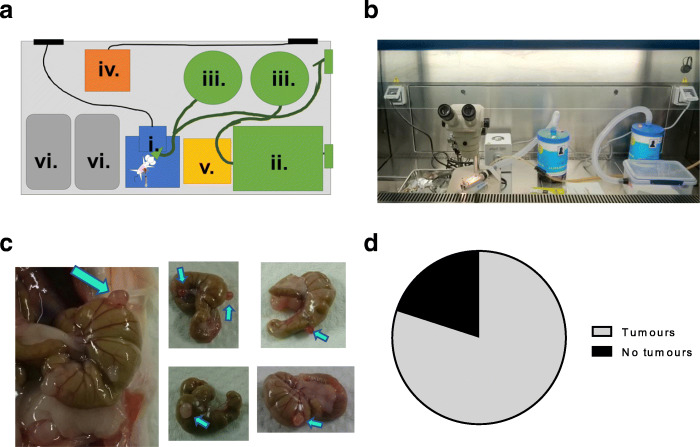
A clinical-grade intracaecal surgery results in intracaecal tumour growth. **a** Surgery set-up in a biological safety cabinet. Surgery is performed on a microscope (i.) to visualise the space between the serosa and subserosa of the caecum. The mouse is anaesthetised in the induction box (ii.) before transfer to the nose cone, both of which are attached to gas scavengers (iii.) to remove waste gas. A hot bead steriliser (iv.) is used to sterilise instrument tips between mice. A non-sterile area (v.) contains pre-surgery prep items and a sterile area (vi.) contains tools and disposables for use during surgery. **b** Photo of in cabinet set-up. **c** Photos of developed intracaecal CT26 tumours on the caecum as indicated by arrows. Some mice grew multiple tumours, as indicated by multiple arrows. **d** Frequency of mice that received intracaecal CT26 cell injection which developed detectable tumours which could be analysed by flow cytometry, *n* = 15

In order to study the effect of a physiologically relevant tumour site, we used an intracaecal surgical model of CRC, modified from the study by Tseng et al. Figure 2 shows an overview of the process used to limit introducing variability and changes in immune response during the procedure. For example, working in a biological safety cabinet can eliminate most potential airborne contamination by maintaining the sterility of the equipment, materials, and space inside the hood through air recirculation and provide a physical barrier between the environment and the mouse. (Fig. 2a, b).

Images of representative tumours are shown in Fig. 2c. The frequency of mice that grew tumours is shown in Fig. 2d, indicating that this method of administration is less consistent than subcutaneous injections commonly used to model cancer. A detailed workflow of all considerations used in developing this model are shown in Additional File 5.

### Tumour infiltrating immune cell populations differ in mice receiving intracaecal versus subcutaneous tumours

**Fig. 3:**
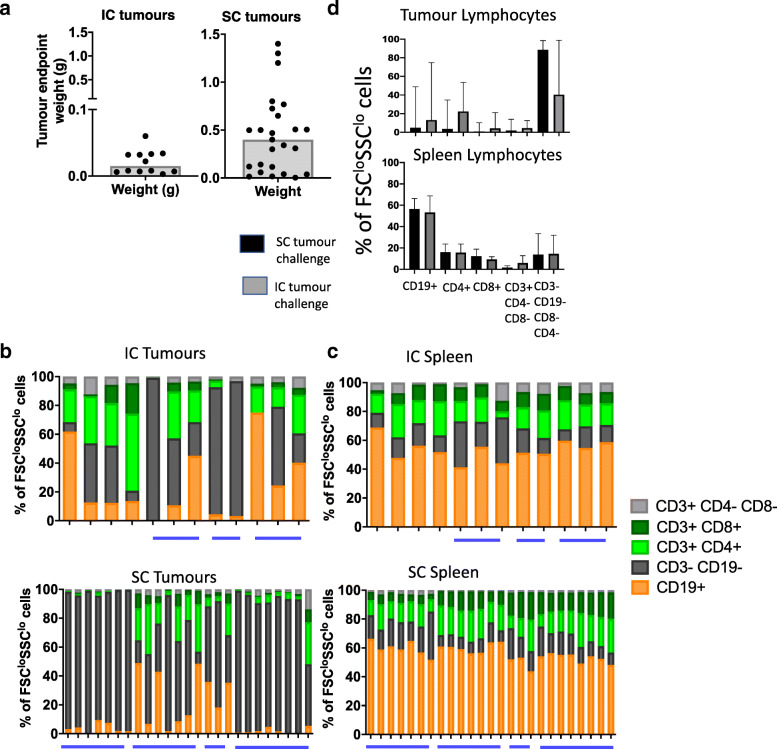
T cells are the dominant lymphocytes in IC CT26 tumours while B cells are the dominant lymphocyte in SC CT26 tumours. **a** Endpoint weight of tumours injected intracaecally (IC) or subcutaneously (SC). If multiple tumours grew, these were combined and weighed together. Each data point represents tumour weight from an individual mouse. Data are shown with median. **b** Frequencies of lymphocyte populations in intracaecal (top graph) and subcutaneous (bottom graph) CT26 tumours. **c** Frequencies of lymphocyte populations in the spleens of mice injected IC (top graph) or SC (bottom graph) with CT26 tumours. Frequencies were normalised to 100. Each bar represents an individual mouse. IC tumours and spleens 1–4 were collected and analysed separately. All other tumours were collected and analysed in batches, as indicated by the blue bars. **d** Pooled data from all mice in all experiments shown in B and C; median +/− SD. IC *n* = 12, from 7 individual experiments. SC *n* = 25, from 4 individual experiments

We chose the CT26 colorectal adenocarcinoma model to further study the impact of injection site on immune infiltrate. Mice were injected either subcutaneously or intracaecally and infiltrating immune cells in spleen, lymph nodes and tumours quantified. Figure 3a shows the size of the tumours at the point of collection – because subcutaneous tumours can be measured non-invasively, they are able to grow to a larger size before reaching ethical endpoints. The intracaecal tumour could not be monitored non-invasively, and so we have used a previously determined ethically sound date post injection [5]. For this reason, we have only one time point of tumour size and the size is different between intracaecal and subcutaneous tumours, therefore, data are shown as percent of lymphoid or myeloid cells (corresponding graphs of absolute numbers are shown in Additional Files 6 and 7).

**Fig. 4:**
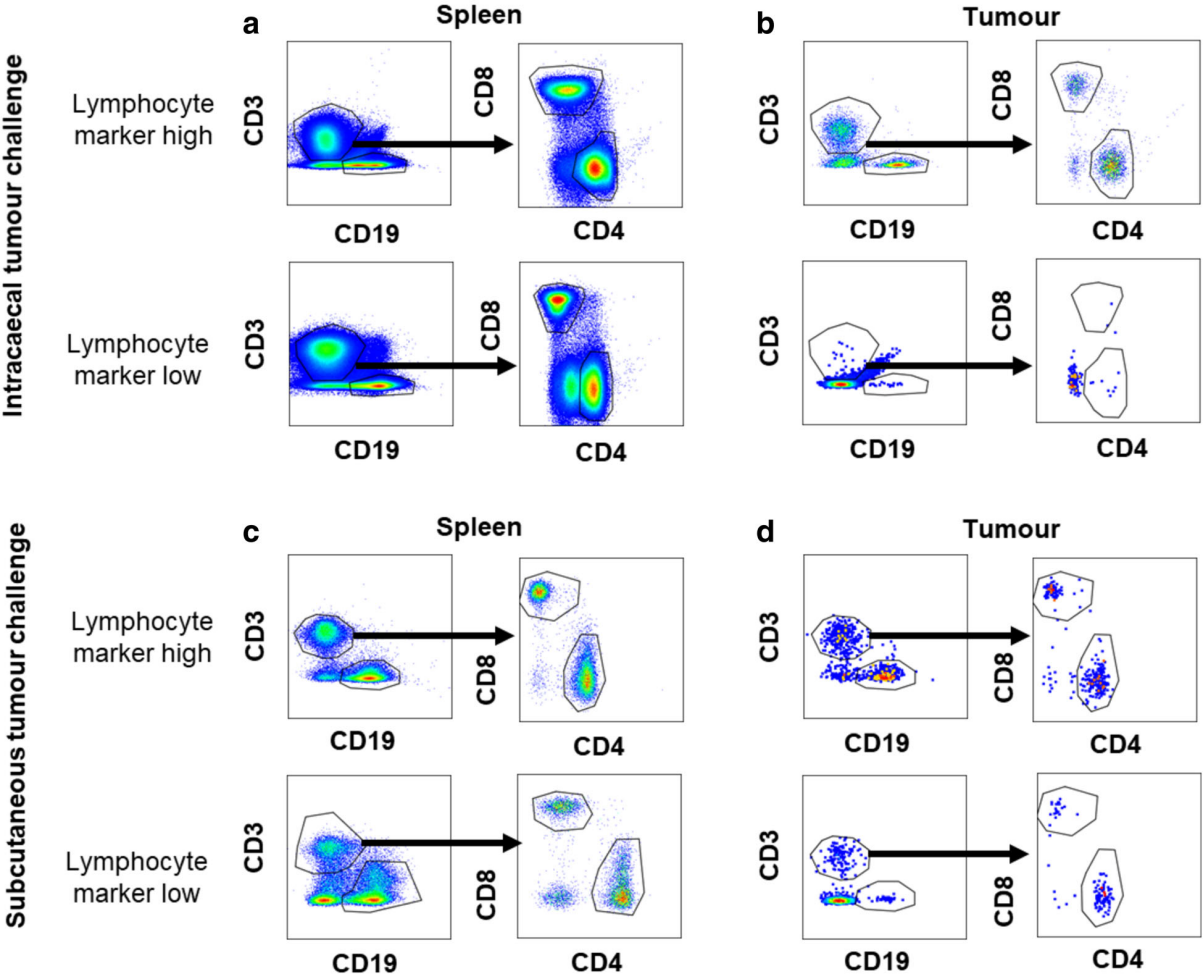
Representative flow cytometry plots from samples with high versus low lymphocyte number from IC and SC tumours and spleens. **a** Representative plots showing high (top plots) and low (bottom plots) lymphocyte number in spleen and **b** tumour samples from mice challenged with IC CT26 tumours. **c-d** As for a and b but in mice challenged with SC CT26 tumours

Figure 3b shows the proportion of T cell subsets in the tumours of individual mice injected intracaecally (top) or subcutaneously (bottom). The graphs represent cells gated by size as “lymphocytes” then serially gated on CD3/CD19 to define T and B cells, respectively, then the CD3+ cells are divided into CD4+, CD8+ or CD4-CD8- (Additional File 3). CD335 was included in a subset of intracaecal tumours and was expressed in approximately half of the CD3-CD19- cells, indicating that these cells may be NK cells (Additional File 8). Our analysis was deliberately chosen to show that selecting known populations may mean that other cells are not counted during these types of analyses, and that in some cases they represent a significant fraction of the tumour infiltrate. We chose to present the frequency of infiltrating cells, rather than the absolute number because the number of infiltrating cells was variable within the tumour (Additional Files 6 and 7 show absolute numbers) We saw a higher frequency of CD3-CD19- cells (putative NK cells) in subcutaneous tumours and a higher frequency of CD3+ CD4+ cells in intracaecal tumours. The presence of CD4-CD8- T cells may indicate downregulation of the coreceptor due to an immunosuppressive environment or a defective T cell population as previously described [4], Blue horizontal bars represent different experiments. From these data, it is clear that the tumour infiltrate can vary between experiments and, importantly, also between individual mice within the same experiment. In contrast, Fig. 3c shows the same information for the spleens of the matching experiment and shows that these populations are consistent across both experiments and mice. Figures 3d shows the same data as for 3b-c, but presented as pooled data with median and standard deviation, emphasising the importance of presenting the true variability of experiments**.** Figure 4 shows representative flow cytometry plots of high versus low tumour infiltrating T cells, highlighting the problems of interpreting changes in frequencies of immune populations across different samples. Together, these data demonstrate the variability in lymphocyte recruitment in individual mice and also show that subcutaneous tumours are immunologically distinct from intracaecal tumours.

**Fig. 5:**
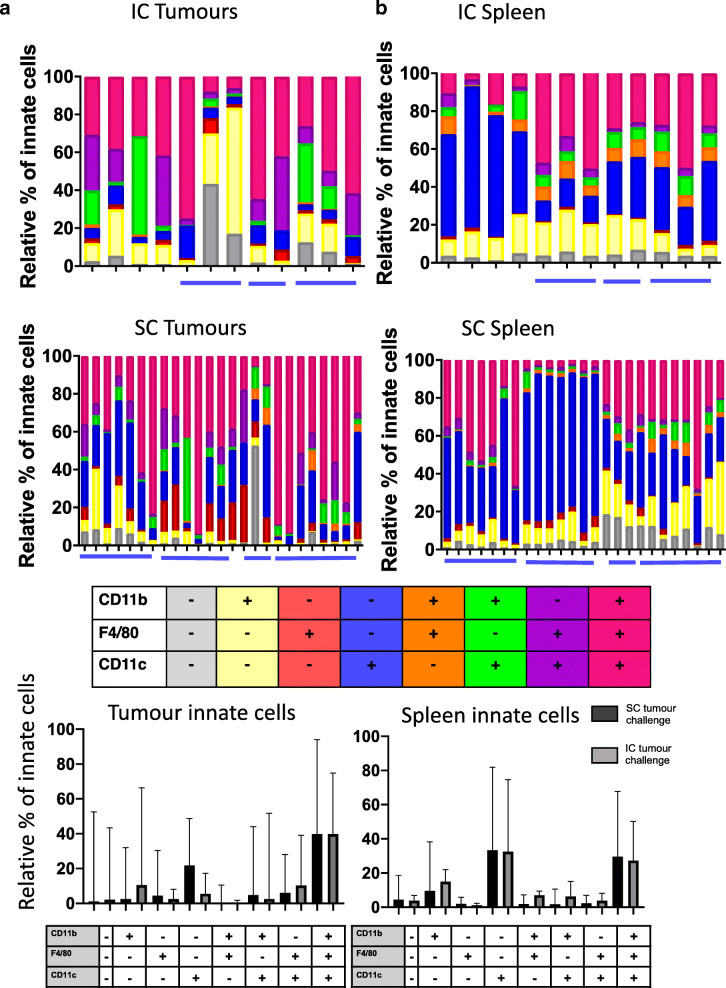
Myeloid cells are CD11b+ CD11c+ and F4/80+ in both IC and SC tumours but there is heterogeneity in spleen cell frequencies. **a** Frequency of myeloid cell populations in in intracaecal (top graph) and subcutaneous (bottom graph) CT26 tumours. Frequencies were normalised to 100. Each bar represents an individual mouse. **b** Frequencies of myeloid cell populations in the spleens of mice challenged IC (top graph) or SC (bottom graph) with CT26 tumours. Frequencies were normalised to 100. Each bar represents an individual mouse. IC tumours and spleens 1–4 were collected and analysed separately. All other tumours were collected and analysed in batches, as indicated by the blue bars. **c** Pooled data from all mice in all experiments shown in a and b; median +/− SD. IC *n* = 12, from 7 individual experiments. SC *n* = 25, from 4 individual experiments

Our second analyses were focussed on myeloid cells (Fig. 5). We used three markers – CD11b, CD11c and F480 to cover a spectrum of macrophage and dendritic cell populations [6, 7] – data are represented by a Boolean gating approach for all three markers (see key and Additional File 4). We saw a higher frequency of F4/80+ cells in subcutaneous tumours than in intracaecal tumours. Similar to the lymphocyte populations, there was variability both between experiments and between individual mice within each experiment. There were more clear differences in cell infiltrate when comparing intracaecal tumours with subcutaneous tumours, again indicating that these two sites are not immunologically representative of each other. Figures 5c shows the same data as for 3b-c, but presented as pooled data with median and standard deviation, emphasising the importance of presenting the true variability of experiments**.** In contrast to lymphoid cells, the myeloid populations in both the spleen and the tumour were also different following intracaecal versus subcutaneous injection, indicating that the systemic as well as the local response may be altered depending on tumour site.

## Discussion

To successfully model human cancer in a mouse model, it is imperative that the immune cells infiltrating the tumour are similar in subset composition and percentage of infiltrating cells to human disease. A model that closely mimics human disease should provide information on cancer progression and illustrate any potential effects of cancer therapeutics. In this study, we compared two variables – mouse strain with tumour model, and the site of injection. Unsurprisingly, there were differences in the infiltrate of immune cells in the tumours, and lymphoid organs of mice receiving different tumour cell lines. However, the infiltrate differences were much more apparent when using the same tumour cell line delivered via subcutaneous versus intracaecal injection. We saw a higher frequency of CD3-CD19- (putative NK) cells and F4/80+ cells in subcutaneous tumours and a higher frequency of CD3+ CD4+ cells in intracaecal tumours. We identified a large population of CD3+ CD4- CD8- T cells infiltrating the tumours of both the B16-OVA and CT26 models, as has previously been published. These cells have been described as suppressive and may explain the growth of the tumours, rather than inhibition [4]. Surprisingly, both tumours had a similar frequency of F4/80+ CD11b+ CD11c+ cells, suggesting these cells were less sensitive to tissue-specific factors. The myeloid cell populations in the spleen were also different when mice were given tumours intracaecally versus subcutaneously – without further functional study, it is difficult to comment on the reasons for this difference, however, our research highlights the variability both within experiments and between individual mice when studying a complex disease like cancer.

The T cell infiltrate is an important indicator of patient response to tumours, especially CRC. A high infiltrate of T cells, including CD3+, CD4+, and CD8+ T cells, in the tumour corresponds to increases in disease free survival and overall survival in CRC patients [8]. In the context of cancer vaccination in mouse models, our previous work has indicated that T cells can provide vaccine-generated protection [5]. The differences seen in lymphocyte infiltration in both mouse models of cancer and between injection sites of the same tumour, demonstrate that establishing a “baseline” readout for a model of cancer is essential before testing interventions. We did not study functional data in this work, but analysis of cytotoxic ability and cytokine profiles is likely to be important in establishing a relevant model for human disease.

Key myeloid cells involved in cancer progression are APCs, including DCs and macrophages, such as tumour associated macrophages (TAMs) [3, 9]. Infiltration of mature TAMs into the tumour has been shown to increase patient survival and disease prognosis [10–12]. However, other studies have shown that TAMs can promote tumour growth [13–15] through the production of proinflammatory cytokines, growth factors, and proteolytic enzymes, the stimulation of angiogenesis, and the remodelling of the extracellular matrix, leading to an immunosuppressive environment [16]. Because of the lack of a clear conclusion on the effect of innate cell infiltration on patient survival, including the role of myeloid derived suppressor cells, these cells are important to monitor during therapeutic testing. Our work showed differences in the myeloid compartment in both the tumour and the spleen when tumour cells were administered subcutaneously versus intracaecally. After intracaecal injection there was a higher frequency of F480+ CD11c+ cells in the spleens compared to subcutaneous injection and high variability within both sets of tumours. These differences are likely confounded by the small number of cells present in each tissue, and over interpretation of data in this context is possible.

A caveat to this work is that intracaecal tumour growth cannot easily be monitored over time, and therefore an ethical experimental endpoint was predetermined and used. Despite the strengths of using an orthotopic mouse model, achieving consistency in tumour growth was more difficult than in subcutaneous tumours. Further work using luminescent tumour cell lines and in vivo imaging will allow the monitoring of intracaecal tumour growth and changes in immune response over time could be more accurately measured.

## Conclusions

In this study, we showed that the analysis of immune infiltrates in mouse models of cancer are likely to be confounded by 1) significant experimental variability between and within experiments, leading to difficulties in interpreting complex immune data; 2) analysis of individual “known” cells, such as CD3+ CD4+ cells, based on predetermined phenotype that may miss large populations of unconventional cells, such as CD3+ CD4- CD8- cells; and 3) differences in tumour type or injection site that can have large effects on immune infiltrate composition. The results demonstrate the importance of showing baseline data in research and to take a cautionary approach in translating preclinical data into human research.

## Supplementary Information

**Additional file 1.** Table of flow cytometry antibodies

**Additional file 2.** Table of flow cytometry antibodies

**Additional file 3 **Representative gating strategy for identifying lymphocyte and innate cell populations. Single cell suspensions from each tissue were gated as follows: **A.** Time gate. **B.** Separating SSC-A and FSC-A low lymphocytes (bottom gate) and SSC-A and FSC-A high myeloid cells (top gate). **C.** Live lymphocytes **D.** Single lymphocytes **G.** CD3+ CD19- T cells and CD3- CD19+ B cells. **H.** T cells were further gated into CD4+ or CD8+. **E.** Live myeloid cells. **F.** CD3- CD19- gate to remove non-myeloid cells. **I.** Single myeloid cells. **J.** Myeloid cells where split based on CD11b and F480 expression before being further stratified based on CD11c expression (**K-N.**). Gates were determined using previously established gating strategies and FMOs (see Additional File 4). Data are from one representative spleen sample from a mouse challenged with IC CT26 tumour cells.

**Additional file 4 **Representative myeloid cell gating strategy based on FMOs in an IC spleen sample. **A.** CD11b FMO. **B.** F480 FMO. **C.** FMOs were used to separate myeloid cells into quadrants: CD11b- F480-, CD11b + F480-, CD11b- F480+, and CD11b + F480+. From these quadrants, cells were then split into CD11c + or CD11c- (**D.**). **E.** CD11c FMO for each quadrant.

**Additional file 5.** Alterations to ensure intracaecal surgery is performed in sterile conditions. The two main areas of optimisation that were addressed included the physical restraints of working in an enclosed biological safety cabinet and using sterile equipment.

**Additional file 6 **Lymphocyte cell counts in the tumours and spleens of mice that received intracaecal (IC) or subcutaneous (SC) CT26 tumours. Cells from processed tissue were stained and live cells identified using trypan blue exclusion, giving the total live cell count for the tissue sample. This total live cell count was used in combination with the frequency of each subset out of total live cells (calculated using FlowJo analysis) to generate the cell number of each subset. **A.** Number of lymphocytes in IC (top graph) and SC (bottom graph) CT26 tumours. **B.** Number of lymphocytes in the spleens of mice injected with IC (top graph) or SC (bottom graph) CT26 tumours. IC *n* = 12, pooled from 7 individual experiments. SC *n* = 25, pooled from 4 individual experiments. Each data point represents a tissue sample from an individual mouse.

**Additional file 7 **Myeloid cell counts in the tumours and spleens of mice that received intracaecal (IC) or subcutaneous (SC) CT26 tumours. Cells from processed tissue were stained and live cells identified using trypan blue exclusion, giving the total live cell count for the tissue sample. This total live cell count was used in combination with the frequency of each subset out of total live cells (calculated using FlowJo analysis) to generate the cell number of each subset. **A.** Number of myeloid cells in IC (top graph) and SC (bottom graph) CT26 tumours. **B.** Number of myeloid cells in the spleens of mice injected with IC (top graph) or SC (bottom graph) CT26 tumours. IC *n* = 12, pooled from 7 individual experiments. SC *n* = 25, pooled from 4 individual experiments. Each data point represents a tissue sample from an individual mouse.

**Additional file 8 **Approximately half of CD3- CD19- lymphocytes are CD335+ NK cells. **A-C.** Samples were gated as in Additional File 3 to the CD3 and CD19 gate, and then gated on CD335. Representative plots are shown for the **A.** lymph nodes, **B.** spleens, and **C.** tumours from mice challenged intracaecally with tumour. **D.** Graph of frequencies of CD335+ lymphocytes gated as in **A-C.** Each data point represents an individual mouse. Data are shown with the median.

## Data Availability

The datasets generated and/or analysed during the current study are available from the corresponding author on reasonable request.

## Abbreviations

APC^min^: Adenomatous polyposis coli
CRC: Colorectal cancer
DC: Dendritic cell
I.C.: Intracaecal
OVA: Ovalbumin
S.C.: Subcutaneous
TAM: Tumour associated macrophage
TME: Tumour microenvironment
