# Supplementary material for: Extensive variability in the composition of immune infiltrate in different mouse models of cancer
Source: Lab Anim Res. 2020 Nov 19;36:43. doi: 10.1186/s42826-020-00075-9 (PMC7678281; doi:10.1186/s42826-020-00075-9)
Supplement: Supplementary file 2 — Additional file 2. Table of flow cytometry antibodies [file 42826_2020_75_MOESM2_ESM.pdf]

Antibody	Fluorophore/dye	Clone	Dilution	Supplier
**CD3**	FITC	17A2	1:800	Biolegend
**CD4**	APC-H7	GK1.5	1:400	Becton Dickinson
**CD8**	APC	53-6.7	1:400	Biolegend
**CD19**	BB700	1D3	1:400	Becton Dickinson
**CD11c**	BV421	N418	1:400	Becton Dickinson
**CD11b**	AF700	M1/70	1:800	Becton Dickinson
**F480**	PECy5	BM8	1:200	Biolegend
**CD335**	PECy7	29A1.4	1:400	Biolegend
**Fixable red**	Texas red	-	1:1000	Life Technologies

Additional File 2 – Flow cytometry panel
