# Supplementary material for: Extensive variability in the composition of immune infiltrate in different mouse models of cancer
Source: Lab Anim Res. 2020 Nov 19;36:43. doi: 10.1186/s42826-020-00075-9 (PMC7678281; doi:10.1186/s42826-020-00075-9)
Supplement: Supplementary file 3 — Additional file 3 Representative gating strategy for identifying lymphocyte and innate cell populations. Single cell suspensions from each tissue were gated as follows: A. Time gate. B. Separating SSC-A and FSC-A low lymphocytes (bottom gate) and SSC-A and FSC-A high myeloid cells (top gate). C. Live lymphocytes D. Single lymphocytes G. CD3+ CD19- T cells and CD3- CD19+ B cells. H. T cells were further gated into CD4+ or CD8+. E. Live myeloid cells. F. CD3- CD19- gate to remove non-myeloid cells. I. Single myeloid cells. J. Myeloid cells where split based on CD11b and F480 expression before being further stratified based on CD11c expression (K-N.). Gates were determined using previously established gating strategies and FMOs (see Additional File 4). Data are from one representative spleen sample from a mouse challenged with IC CT26 tumour cells. [file 42826_2020_75_MOESM3_ESM.pdf]

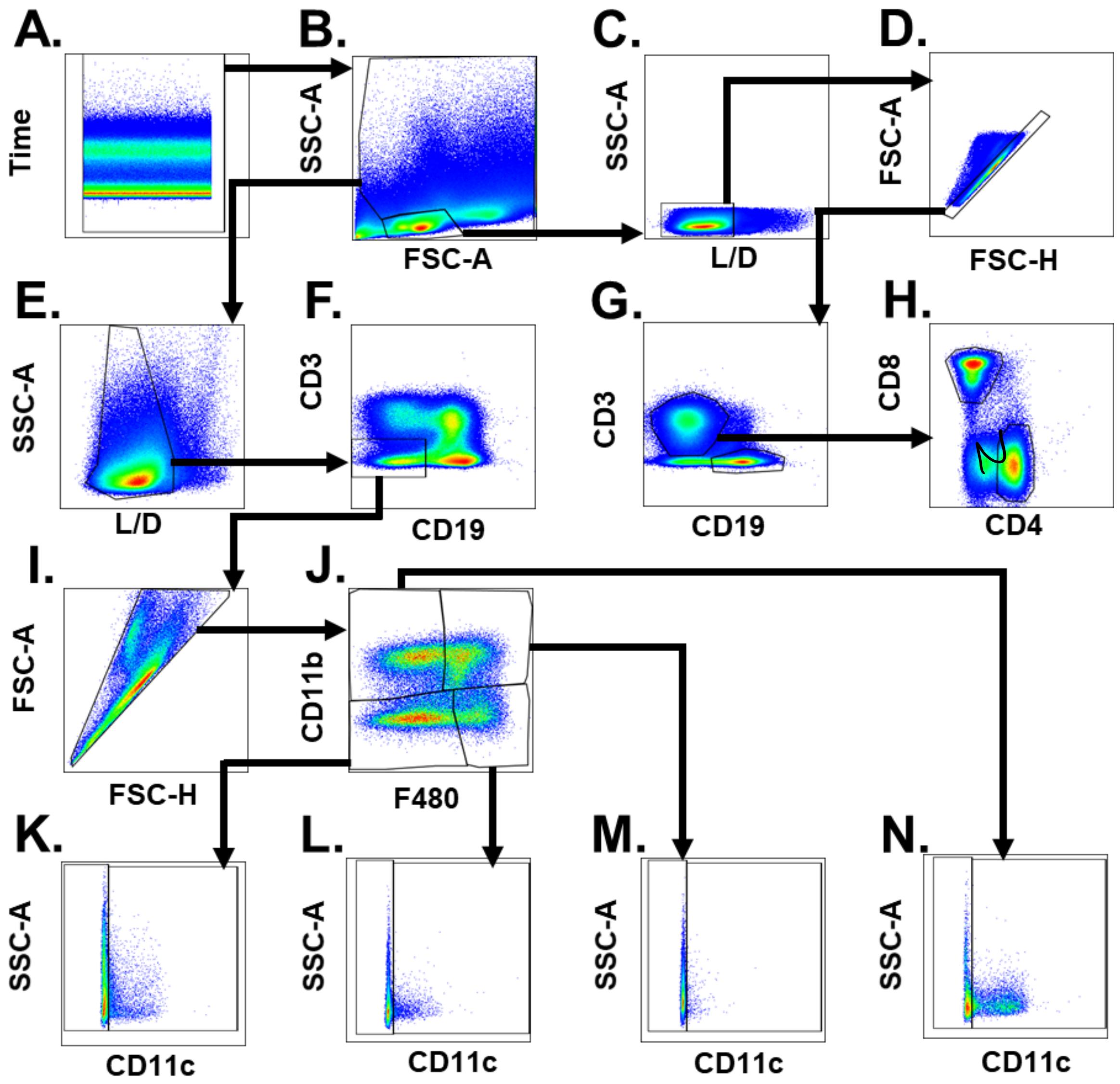

Additional File 3: Representative gating strategy for identifying lymphocyte and innate cell populations. Single cell suspensions from each tissue were gated as follows: **A.** Time gate. **B.** Separating SSC-A and FSC-A low lymphocytes (bottom gate) and SSC-A and FSC-A high myeloid cells (top gate). **C.** Live lymphocytes **D.** Single lymphocytes **G.** CD3+ CD19- T cells and CD3- CD19+ B cells. **H.** T cells were further gated into CD4+ or CD8+. **E.** Live myeloid cells. **F.** CD3- CD19- gate to remove non-myeloid cells. **I.** Single myeloid cells. **J.** Myeloi cells where split based on CD11b and F480 expression before being further stratified based on CD11c expression (**K-N.**). Gates were determined using previously established gating strategies and FMOs (see **Supplementary Figure 2**). Data are from one representative spleen sample from a mouse challenged with IC CT26 tumour cells.
