# Supplementary material for: Extensive variability in the composition of immune infiltrate in different mouse models of cancer
Source: Lab Anim Res. 2020 Nov 19;36:43. doi: 10.1186/s42826-020-00075-9 (PMC7678281; doi:10.1186/s42826-020-00075-9)
Supplement: Supplementary file 4 — Additional file 4 Representative myeloid cell gating strategy based on FMOs in an IC spleen sample. A. CD11b FMO. B. F480 FMO. C. FMOs were used to separate myeloid cells into quadrants: CD11b- F480-, CD11b + F480-, CD11b- F480+, and CD11b + F480+. From these quadrants, cells were then split into CD11c + or CD11c- (D.). E. CD11c FMO for each quadrant. [file 42826_2020_75_MOESM4_ESM.pdf]

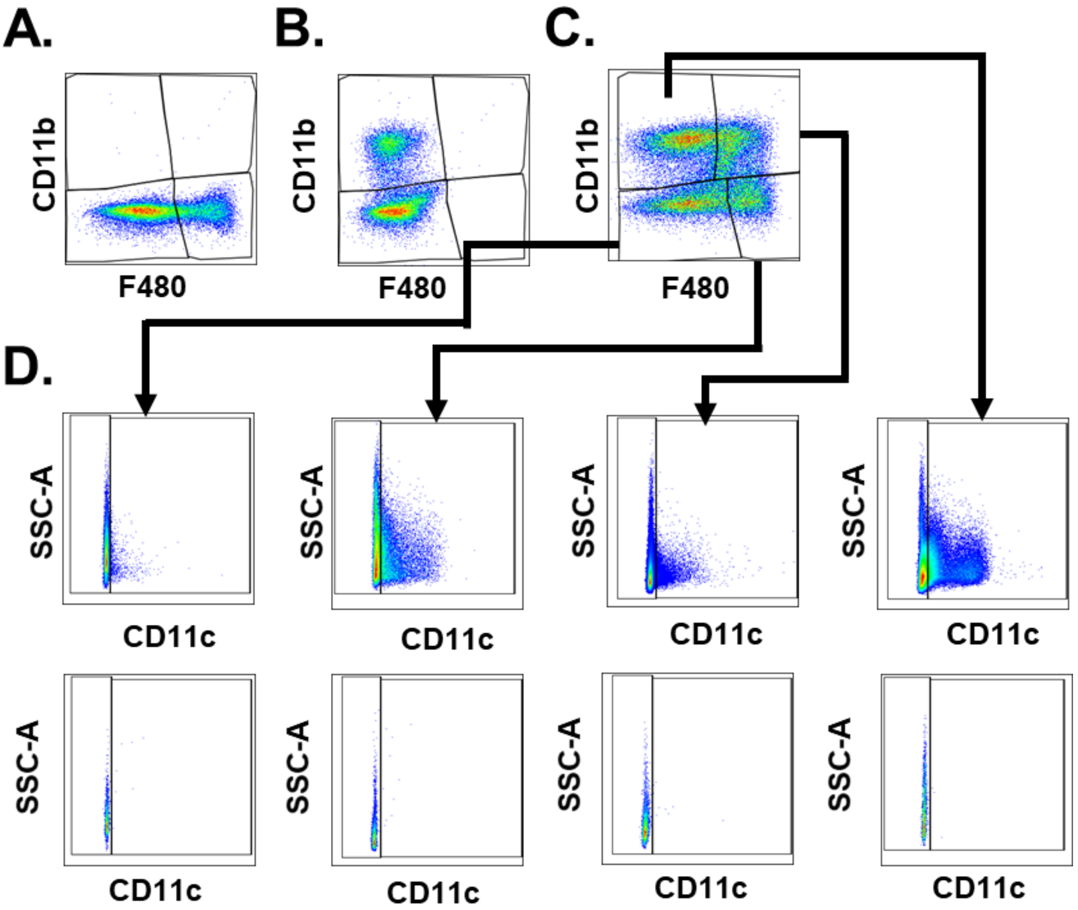

Additional File 4: Representative myeloid cell gating strategy based on FMOs in an IC spleen sample. **A.** CD11b FMO. **B.** F480 FMO. **C.** FMOs were used to separate myeloid cells into quadrants: CD11b- F480-, CD11b+ F480-, CD11b- F480+, and CD11b+ F480+. From these quadrants, cells were then split into CD11c+ or CD11c- (**D.**). **E.** CD11c FMO for each quadrant.
