# Supplementary material for: Extensive variability in the composition of immune infiltrate in different mouse models of cancer
Source: Lab Anim Res. 2020 Nov 19;36:43. doi: 10.1186/s42826-020-00075-9 (PMC7678281; doi:10.1186/s42826-020-00075-9)
Supplement: Supplementary file 5 — Additional file 5. Alterations to ensure intracaecal surgery is performed in sterile conditions. The two main areas of optimisation that were addressed included the physical restraints of working in an enclosed biological safety cabinet and using sterile equipment. [file 42826_2020_75_MOESM5_ESM.pdf]

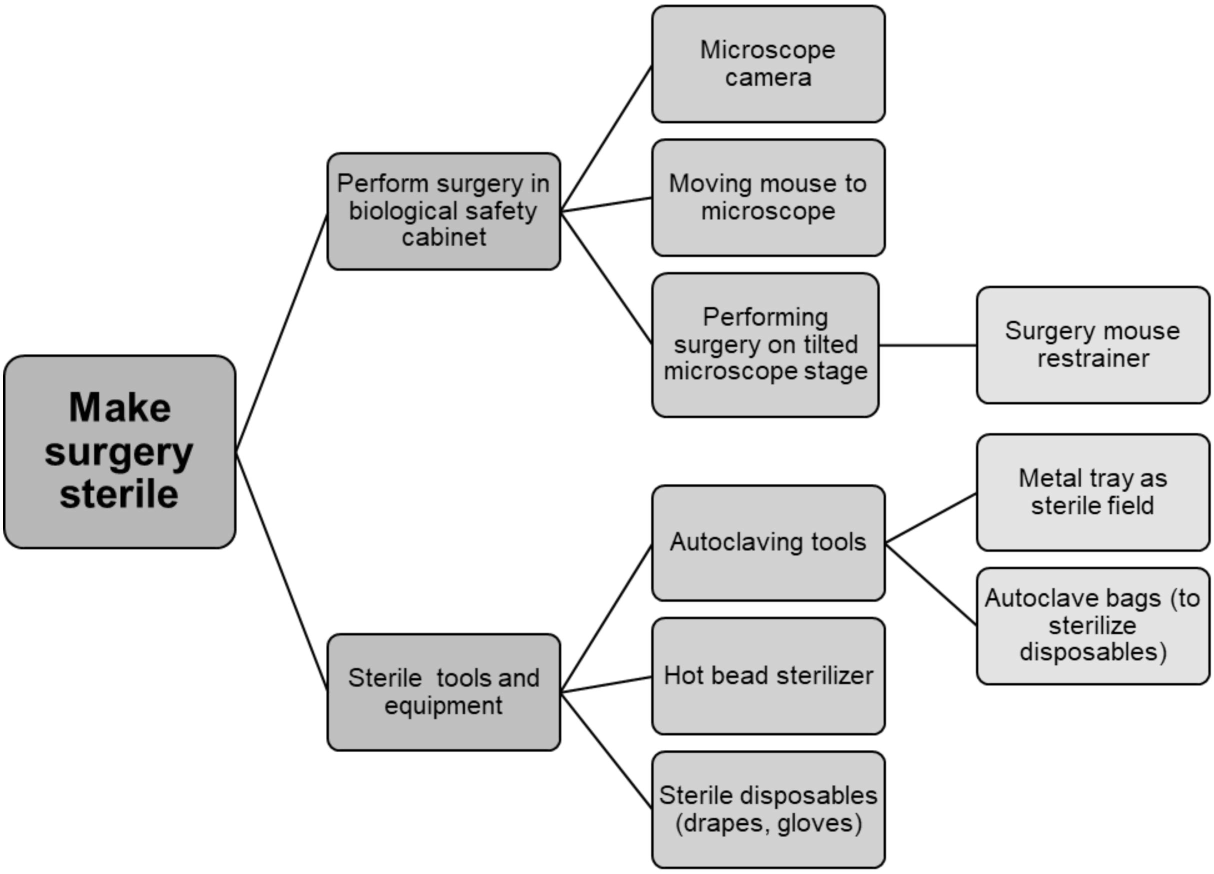

Additional File 5: Alterations to ensure intracaecal surgery is performed in sterile conditions.

The two main areas of optimisation that were addressed included the physical restraints of working in an enclosed biological safety cabinet and using sterile equipment.
