# Supplementary material for: Extensive variability in the composition of immune infiltrate in different mouse models of cancer
Source: Lab Anim Res. 2020 Nov 19;36:43. doi: 10.1186/s42826-020-00075-9 (PMC7678281; doi:10.1186/s42826-020-00075-9)
Supplement: Supplementary file 6 — Additional file 6 Lymphocyte cell counts in the tumours and spleens of mice that received intracaecal (IC) or subcutaneous (SC) CT26 tumours. Cells from processed tissue were stained and live cells identified using trypan blue exclusion, giving the total live cell count for the tissue sample. This total live cell count was used in combination with the frequency of each subset out of total live cells (calculated using FlowJo analysis) to generate the cell number of each subset. A. Number of lymphocytes in IC (top graph) and SC (bottom graph) CT26 tumours. B. Number of lymphocytes in the spleens of mice injected with IC (top graph) or SC (bottom graph) CT26 tumours. IC n = 12, pooled from 7 individual experiments. SC n = 25, pooled from 4 individual experiments. Each data point represents a tissue sample from an individual mouse. [file 42826_2020_75_MOESM6_ESM.pdf]

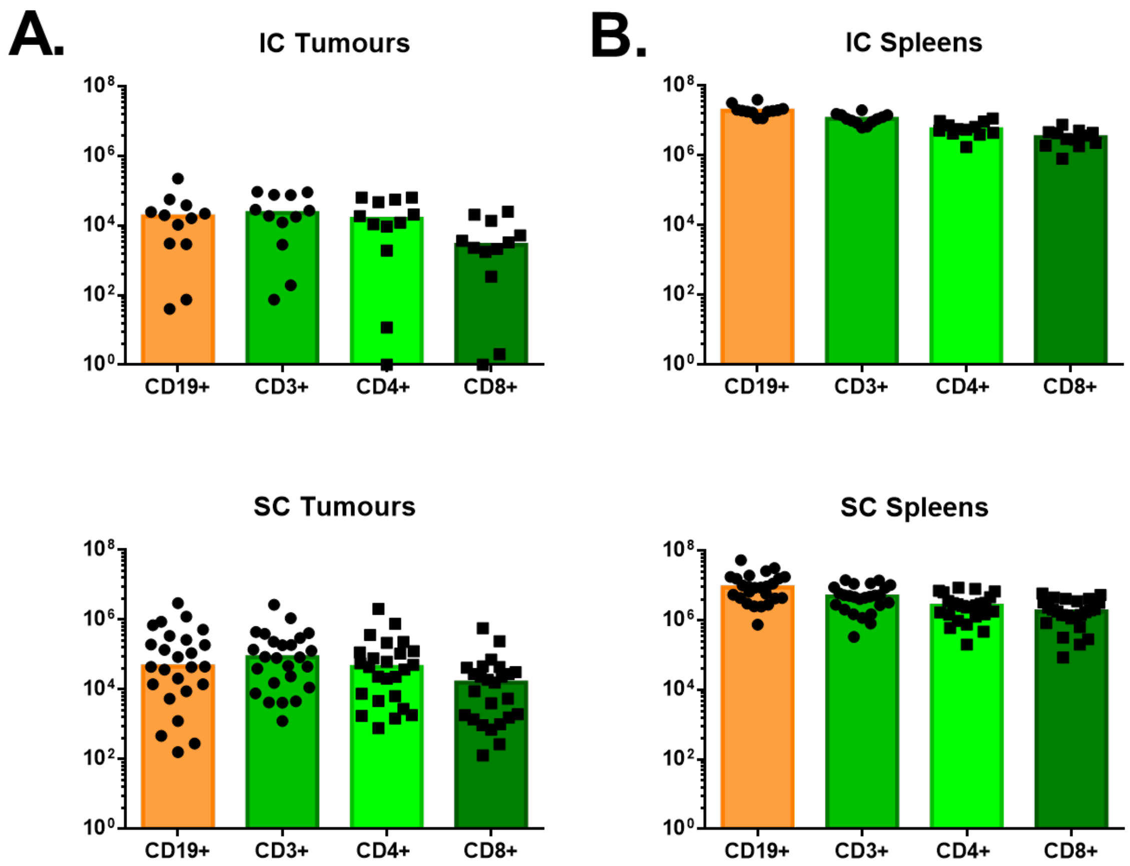

Additional File 6: Lymphocyte cell counts in the tumours and spleens of mice that received intracaecal (IC) or subcutaneous (SC) CT26 tumours. Cells from processed tissue were stained and live cells identified using trypan blue exclusion, giving the total live cell count for the tissue sample. This total live cell count was used in combination with the frequency of each subset out of total live cells (calculated using FlowJo analysis) to generate the cell number of each subset. **A.** Number of lymphocytes in IC (top graph) and SC (bottom graph) CT26 tumours. **B.** Number of lymphocytes in the spleens of mice injected with IC (top graph) or SC (bottom graph) CT26 tumours. IC n = 12, pooled from 7 individual experiments. SC n = 25, pooled from 4 individual experiments. Each data point represents a tissue sample from an individual mouse.
