# Supplementary material for: Extensive variability in the composition of immune infiltrate in different mouse models of cancer
Source: Lab Anim Res. 2020 Nov 19;36:43. doi: 10.1186/s42826-020-00075-9 (PMC7678281; doi:10.1186/s42826-020-00075-9)
Supplement: Supplementary file 8 — Additional file 8 Approximately half of CD3- CD19- lymphocytes are CD335+ NK cells. A-C. Samples were gated as in Additional File 3 to the CD3 and CD19 gate, and then gated on CD335. Representative plots are shown for the A. lymph nodes, B. spleens, and C. tumours from mice challenged intracaecally with tumour. D. Graph of frequencies of CD335+ lymphocytes gated as in A-C. Each data point represents an individual mouse. Data are shown with the median. [file 42826_2020_75_MOESM8_ESM.pdf]

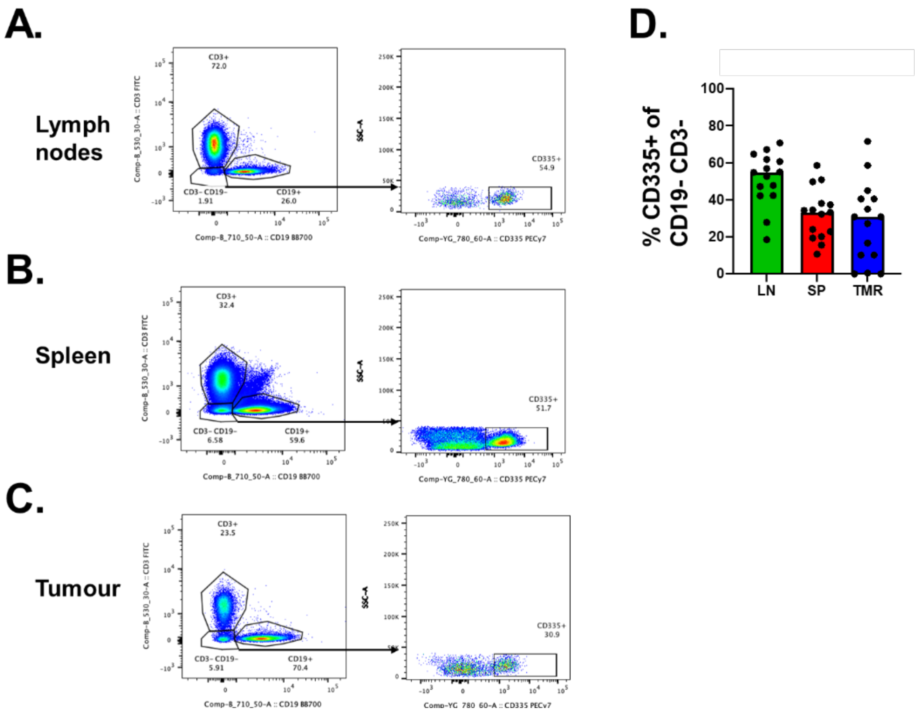

Additional File 8: Approximately half of CD3- CD19- lymphocytes are CD335+ NK cells. A-C. Samples were gated as in Additional File 3 to the CD3 and CD19 gate, and then gated on CD335. Representative plots are shown for the **A.** lymph nodes, **B.** spleens, and **C.** tumours from mice challenged intracaecally with tumour. **D.** Graph of frequencies of CD335+ lymphocytes gated as in **A-C.** Each data point represents an individual mouse. Data are shown with the median.
